# Research hotspots and trends in lung cancer STAS: a bibliometric and visualization analysis

**DOI:** 10.3389/fonc.2024.1495911

**Published:** 2025-01-03

**Authors:** Xiuhua Peng, Hupo Bian, Hongxing Zhao, Dan Jia, Mei Li, Wenhui Li, Pengliang Xu

**Affiliations:** ^1^ Department of Radiology, The First People’s Hospital of Huzhou, Huzhou, China; ^2^ Department of Respiratory Medicine, The First People’s Hospital of Huzhou, Huzhou, China; ^3^ Department of Thoracic Surgery, The First People’s Hospital of Huzhou, Huzhou, China

**Keywords:** lung cancer, STAS, visualization analysis, bibliometric analysis, deep learning

## Abstract

**Purpose:**

This study employed the R software bibliometrix and the visualization tools CiteSpace and VOSviewer to conduct a bibliometric analysis of literature on lung cancer spread through air spaces (STAS) published since 2015.

**Methods:**

On September 1, 2024, a computer-based search was performed in the Web of Science (WOS) Core Collection dataset for literature on lung cancer STAS published between January 1, 2015, and August 31, 2024. VOSviewer was used to visually analyze countries, institutions, authors, co-cited authors, and keywords, while CiteSpace was utilized to analyze institutional centrality, references, keyword bursts, and co-citation literature. Descriptive analysis tables were created using Excel 2021.

**Results:**

A total of 243 articles were included from the WOS, with a significant increase in annual publications observed since 2018. China, Kadota K, and Fudan University were leading countries, authors, and institutions by publication volume. The top three authors by co-citation count were Kadota K, Chen C, and Adusumilli PS. The journal with the highest publication volume was Lung Cancer, with the most influential journal among the top 10 being the Journal of Thoracic Oncology. The most frequently cited reference was “Lobectomy Is Associated with Better Outcomes than Sublobar Resection in Spread through Air Spaces (STAS)-Positive T1 Lung Adenocarcinoma: A Propensity Score-Matched Analysis.” Keyword clustering categorized the research into four main areas: pathological studies of lung cancer STAS, biological mechanisms, prognostic assessment, and imaging analysis. Current research hotspots include deep learning, lung squamous cell carcinoma, and air spaces STAS.

**Conclusion:**

The current research on lung cancer STAS primarily focuses on pathological studies, biological mechanisms, prognostic assessments, and preoperative imaging model predictions. This study’s findings provide new insights and directions for future research in this area.

**Systematic review registration:**

https://www.crd.york.ac.uk/prospero/#myprospero, identifier 589442.

## Introduction

Lung cancer is the most common and lethal malignant tumor worldwide (1). With the increasing awareness of health check-ups, the application of low-dose CT in early lung cancer screening has gradually increased. Studies have shown that early-stage lung cancer patients can preserve lung function and reduce postoperative complications through sublobar resection. Although surgical treatment significantly improves cure rates and survival, recurrence rates remain as high as 20-30% (2).

In 2015, the World Health Organization first defined spread through air spaces (STAS), referring to tumor cells in specific forms within the surrounding tissue of primary lung cancer. STAS is considered a novel mode of invasion in lung adenocarcinoma (3). STAS is associated with poor prognosis in other types of lung cancer and is a significant risk factor for postoperative local recurrence (4, 5). Performing lobectomy on early lung adenocarcinoma patients with STAS positivity can reduce recurrence rates and improve the 5-year survival rate (6). Therefore, preoperative clarification of STAS status assists surgeons in formulating personalized treatment plans.

Bibliometrics allows quantitative literature analysis to assess research trends and hotspots (7). Software such as VOSviewer and CiteSpace can conduct co-word analysis and co-citation analysis, visually presenting the results. Since 2015, STAS has become a focal point in academic research, attracting interest from researchers worldwide; thus, the literature on STAS has been increasing annually. However, a systematic bibliometric analysis has yet to be conducted. This study aims to summarize past achievements, identify research directions and hotspots, and provide references for future studies.

## Methods

### Data source and literature search strategy

**Figure 1:**
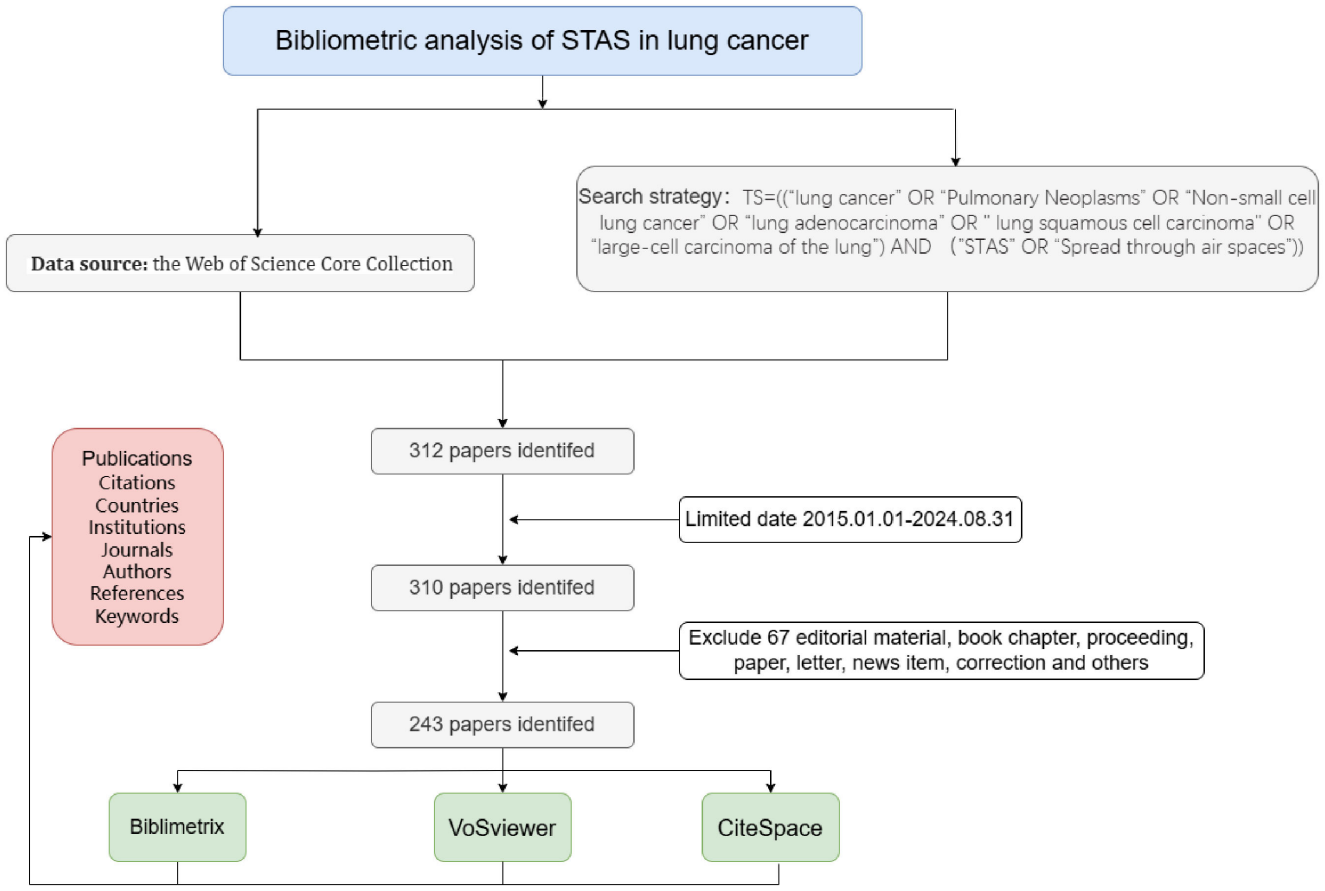
Flowchart of included articles.

We obtained all relevant publications from the Web of Science Core Collection (WOSCC), which includes over 12,000 of the most influential and high-quality scientific journals. The search strategy TS= (“ lung cancer “OR” Pulmonary Neoplasms “OR” Non-small cell lung cancer “OR” lung adenocarcinoma “OR” lung squamous cell carcinoma” OR “large-cell carcinoma of the lung”) AND (“ STAS “OR” Spread through air spaces “)). The timeframe for literature retrieval was set from January 1, 2015, to August 31, 2024, without limiting the range of languages, and the types of literature included were articles and reviews. **Figure 1** illustrates this study’s specific data retrieval techniques and inclusion process. Finally, we exported all retrieved literature and stored it as a text file for subsequent bibliometric analysis.

### Software for bibliometric analysis and visualization analysis

The title, authors, publication year, country/region, institution, keywords, citations, abstract, and references were all sourced from the WOSCC database. The downloaded files were in plain text format. Two researchers utilized VOSviewer version 1.6.20 to extract all data meeting the inclusion criteria for this study, which were then imported into CiteSpace version 6.2.R4 and Microsoft Excel 2019. CiteSpace and VOSviewer were employed for visual analysis in bibliometric research. CiteSpace was used to examine collaboration networks, co-occurrence, and centrality among countries, authors, and institutions. At the same time, VOSviewer was utilized to analyze citation counts and direct link strengths of articles. Instructions for using VOSviewer can be found at https://www.vosviewer.com/getting-started, and CiteSpace can be accessed and downloaded at https://citespace.podia.com/, where relevant text and video tutorials are provided. The R package “Bibliometrix” (version 4.3.3) was also used to analyze differences in publication output among countries.

## Result

### Publication volume and trend analysis

**Figure 2:**
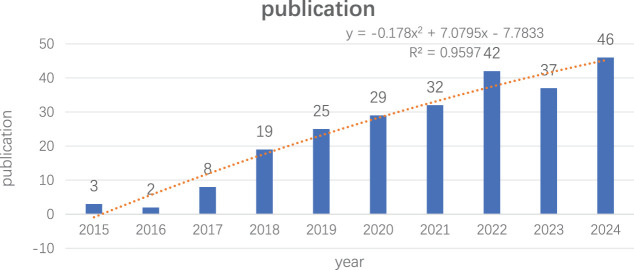
Trends in literature related to lung cancer STAS research from 2015 to 2024.

From 2015 to August 2024, we included 243 articles for analysis following a comprehensive manual screening. The annual publication volume is shown in **Figure 2**. We observed a significant increase in published papers starting in 2017, reaching a peak in 2024 (46 papers). The results of the polynomial fitting analysis indicated an R² value of 0.9597, suggesting that the literature output in this field is expected to continue increasing.

### Analysis of publication countries/regions

**Figure 3:**
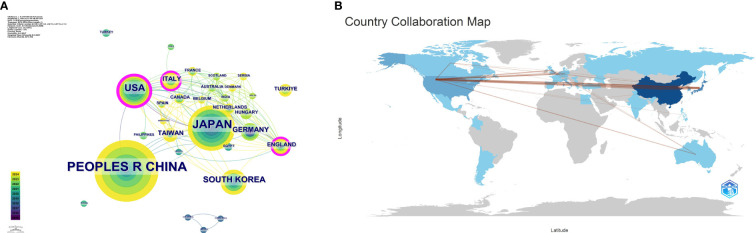
**(A)** Collaboration map among countries/regions. **(B)** Publication volume and collaboration map by country.

These publications originated from 31 countries, with the distribution of the top 10 countries presented in **Table 1**. The total number of publications from the top three countries accounts for the majority (n=211, 86.3%), including the United States from North America (n=33, 13.6%), China from Asia (n=113, 46.5%), and Japan from Asia (n=65, 26.7%). The collaboration network for each country based on publication volume and relationships is illustrated in **Figure 3A**. In CiteSpace, centrality measures the importance of a node as a bridge between other nodes in the network. Specifically, it calculates how many shortest paths a node lies on, reflecting its critical role in information dissemination and network connectivity. Countries with a centrality greater than 0.1 are highlighted with purple circles, including the United States, Italy, and the United Kingdom, indicating their roles as bridges in the flow of research information.

**Table 1:** The Top 10 Countries in Terms of Number of Publications from 2015 to 2024.

Sequence	Country (region)	Publications	Total citations	Average Article Citations	TLS	centrality
1	China	113	1438	12.73	32	0
2	Japan	65	2628	40.43	44	0.09
3	United States	33	1995	60.45	63	0.24
4	South korea	20	330	16.50	11	0
5	Germany	10	504	50.40	18	0.03
6	Italy	9	564	62.67	27	0.13
7	Taiwan,China	8	119	14.88	20	0.01
8	England	6	632	105.33	32	0.14
9	Turkiye	5	2	0.40	0	0
10	Canada	4	528	132.00	20	0.02

### Analysis of publishing institutions

The 243 articles originated from 333 institutions, with China, the United States, and Japan dominating the top ten institutions in research publications. The top three institutions are Fudan University (n=21, 8.6%), Tongji University (n=17, 7.0%), and Harvard University (n=14, 5.8%). Additionally, Harvard Medical School has the highest centrality (**Table 2**). **Figure 3B** shows a global collaborative map. The intensity of color saturation corresponds to the number of articles per country. The thickness of the connecting arrows symbolizes the strength of cooperation between countries.

**Table 2:** The Top 10 Organizations in Terms of Publication from 2015 to 2024.

Sequence	count	centrality	year	Institution	Country
1	21	0.05	2017	Fudan University	China
2	17	0.08	2017	Tongji University	China
3	14	0.03	2016	Harvard University	United States
4	13	0.04	2015	Kagawa University	Japan
5	12	0.11	2016	Harvard Medical School	United States
6	12	0.08	2017	National Cancer Center - Japan	Japan
7	11	0.1	2015	Memorial Sloan Kettering Cancer Center	United States
8	11	0.07	2019	Shanghai Jiao Tong University	China
9	9	0.04	2018	Chinese Academy of Medical Sciences - Peking Union Medical College	China
10	8	0.01	2018	Seoul National University (SNU)	South Korea

### Analysis of publication volume and co-citation in journals

The 243 articles were published in 98 journals, with **Table 3** presenting the journals with the highest publication counts as calculated by VOSviewer. The journal Lung Cancer had the highest number of publications (n=25), followed by the Journal of Thoracic Oncology (n=14) and the American Journal of Surgical Pathology (n=10). Among the top 10 journals, the Journal of Thoracic Oncology had the highest impact factor (IF=21, Q1). During 2018-2019, the Journal of Thoracic Oncology also had the highest citation frequency, with Lung Cancer and the American Journal of Surgical Pathology receiving substantial citations.

**Table 3:** 10 Journals with the Highest Number of Publications from 2015 to 2024.

Sequence	Journal	Publications	Total citations	Average Article Citations	TLS	IF(2023)
1	Lung Cancer	25	456	18.24	434	4.5
2	Journal of Thoracic Oncology	14	1792	128.00	697	21
3	American Journal of Surgical Pathology	10	477	47.70	352	4.5
4	Journal of Thoracic Disease	10	68	6.80	169	2.1
5	Thoracic Cancer	8	113	14.13	160	2.3
6	Annals of Thoracic Surgery	6	229	38.17	200	3.6
7	Frontiers in Oncology	6	49	8.17	113	3.5
8	Interactive Cardiovascular and Thoracic surgery	6	186	31.00	159	1.6
9	Annals of Surgical Oncology	5	67	13.40	69	3.4
10	Cancer Medicine	5	20	4.00	61	2.9

**Figure 4:**
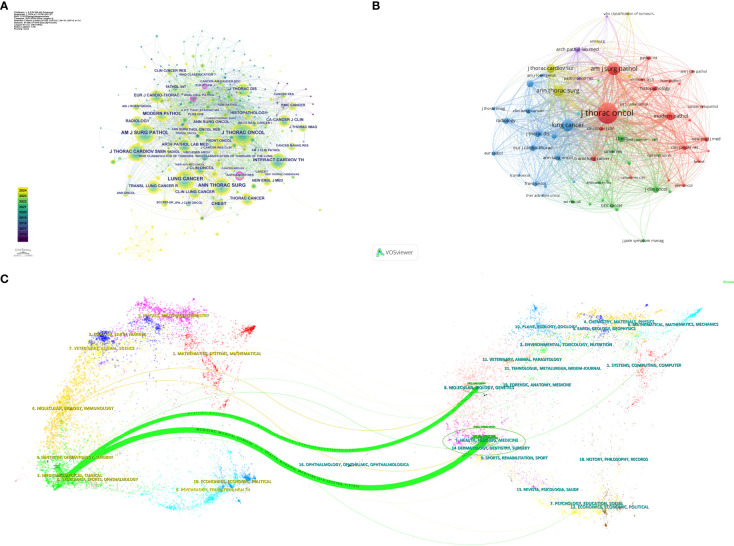
**(A)** Visual map of co-cited journals in the Citespace network. **(B)** Visual map of co-cited journals in the VOSviewer network. **(C)** Overlay of journal dual maps.

A total of 867 co-cited journals were identified from the 243 articles, with **Table 4** showing that the journal with the highest citation count was the Journal of Thoracic Oncology (1497 citations). Co-cited journals with a centrality greater than 0.1 include the European Respiratory Journal, Anticancer Research, and American Journal of Pathology, highlighted with purple circles in **Figure 4A**. **Figure 4A** presents the co-citation knowledge map of journals generated by CiteSpace. **Figure 4B** displays the co-citation knowledge map of journals generated by VOSviewer, where each color represents a distinct cluster, reflecting different research areas or themes derived from the citation relationships among the journals.

**Table 4:** The Top 10 Journals with Total Citations from 2015 to 2024.

Top 10 co-cited journals by citation count, calculated by VOSviewer				Top 10 co-cited journals by betweenness centrality, calculated by Cite Space		
Sequence	Journal	citations	total link strength	Sequence	centrality	Journal
1	Journal of Thoracic Oncology	1497	37286	1	0.14	European Respiratory Journal
2	American Journal of Surgical Pathology	535	15851	2	0.11	Anticancer Research
3	Lung Cancer	461	13814	3	0.1	American Journal Of Pathology
4	Annals of Thoracic Surgery	405	11125	4	0.09	American Journal Of Clinical Pathology
5	Journal Of Thoracic And Cardiovascular Surgery	264	7479	5	0.09	Annal Of Internal Medicine
6	Modern Pathology	209	7965	6	0.08	BMC Cancer
7	Interactive Cardiovascular And Thoracic surgery	157	4461	7	0.08	Plos One
8	Journal Of Clinical Oncology	139	4420	8	0.08	Analytical Cellular Pathology
9	Chest	138	4096	9	0.08	Cancer Cytopathol
10	Journal Of Thoracic Disease	131	4276	10	0.08	Cell

The dual map overlay generated by CiteSpace (**Figure 4C**) illustrates the citation relationships between citing journals and cited journals. The cluster on the left represents citing journals, indicating the forefront of knowledge, while the cluster on the right represents cited journals, reflecting the knowledge base. Each label is centered around the journal’s cluster center and indicates the published articles’ relevant disciplines. Each spline curve starts from a cited journal in the left background and points to a citing journal in the right background. **Figure 4C** shows two prominent citation pathways (in green), indicating that research published in journals within the Health/Nursing/Medicine and Molecular/Biology/Genetics clusters is primarily cited by literature in the Medicine/Medical/Clinical journal cluster, demonstrating a convergence pattern.

### Analysis of authors and co-cited authors

One thousand four hundred thirty-two authors contributed to the 243 articles, the most prolific author being Kadota K. In 2015, Kadota K published the paper titled “Tumor Spread through Air Spaces is an Important Pattern of Invasion and Impacts the Frequency and Location of Recurrences after Limited Resection for Small Stage I Lung Adenocarcinomas” in the Journal of Thoracic Oncology, which established STAS as a significant risk factor for recurrence in the limited resection treatment of non-small cell lung cancer and formally recognized it as a new mode of invasion for lung adenocarcinoma, achieving an impact factor of 21.0. **Table 5** lists the top 10 authors by publication volume.

**Table 5:** The Top 10 Authors in Terms of Publication Volume and Total Citation Times from 2015 to 2024.

Sequence	count	centrality	year	authors
1	13	0.06	2015	Kadota K
2	11	0	2017	Chen C
3	9	0.02	2015	Adusumilli PS
4	9	0	2017	Chen D
5	8	0.01	2015	Travis WD
6	7	0.03	2016	Mino-kenudson M
7	7	0	2018	Chen Y
8	6	0.01	2017	Eguchi T
9	6	0	2018	Cho S
10	6	0	2017	Haba R

**Figure 5:**
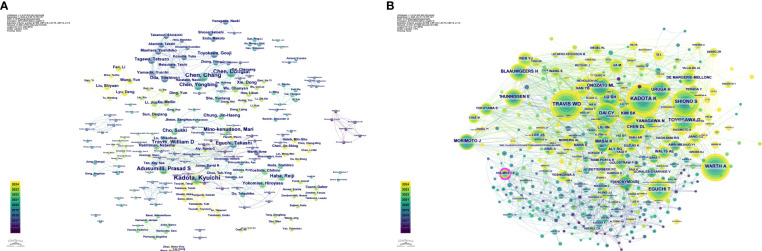
Visualization map of the CiteSpace network among authors **(A)** and among co-cited authors **(B)**.

Additionally, Kadota K had a centrality value of 0.06 in 2015, ranking first and indicating a high level of influence and core status within the academic network. This suggests that Kadota K has a high citation frequency and educational impact. Furthermore, authors such as Mino-Kenudson M and Adusumilli PS exhibited high centrality values in their respective years, reflecting their core status and academic contributions in the relevant fields. **Figure 5A** depicts the detailed visualization map of the CiteSpace network among authors, with each node representing an author. Larger nodes indicate a higher number of publications, while nodes with a yellower color signify more recent activity.

Among the references of the 243 articles, 2,747 authors were co-cited (**Table 6**), with Kadota K leading in citation frequency at 202 citations. However, Holmes CE held the highest centrality value (0.11), indicating an important connecting role between different research groups and influencing knowledge flow across disciplines. Additionally, **Figure 5B** reveals the relationships among authors who are frequently co-cited together.

**Table 6:** Top 10 Co-Cited Authors by Number of Publications and Betweenness Centrality.

Sequence	count	centrality	year	authors	Sequence	count	centrality	year	authors
1	202	0.01	2015	Kadota K	1	24	0.11	2016	Holmes CE
2	173	0.01	2015	Travis WD	2	51	0.09	2016	Anonymous
3	148	0.01	2017	Warth A	3	21	0.09	2017	Detterbeck FC
4	128	0.02	2017	Shino S	4	19	0.09	2017	Kameda K
5	111	0	2018	Toyokawa G	5	14	0.09	2015	Aberle DR
6	106	0.06	2017	Dai CY	6	28	0.08	2019	Moreira AL
7	98	0.03	2017	Eguchi T	7	31	0.07	2019	Yokoyama S
8	89	0.02	2015	Onozato ML	8	29	0.07	2017	Bains S
9	85	0.01	2017	Lu SH	9	25	0.07	2016	Hung JJ
10	74	0.05	2017	Blaauwgeers H	10	4	0.07	2017	Taura T

### Analysis of cited references

Using CiteSpace, the ten most cited articles were identified (**Table 7**). Co-citation refers to the occurrence of two references in the reference list of a third article, establishing a co-citation relationship. The article titled “Lobectomy Is Associated with Better Outcomes than Sublobar Resection in Spread through Air Spaces (STAS)-Positive T1 Lung Adenocarcinoma: A Propensity Score-Matched Analysis” received the highest number of citations, followed by “Tumor Spread through Air Spaces Affects the Recurrence and Overall Survival in Patients with Lung Adenocarcinoma >2 to 3 cm” and “Tumor Spread through Air Spaces is an Important Pattern of Invasion and Impacts the Frequency and Location of Recurrences after Limited Resection for Small Stage I Lung Adenocarcinomas.” All of these articles were published in the Journal of Thoracic Oncology.

**Table 7:** References with the Top 10 Cited Times.

Sequence	count	centrality	year	cited references
1	89	0.03	2019	Lobectomy Is Associated with Better Outcomes than Sub lobar Resection in Spread through Air Spaces (STAS)-Positive T1 Lung Adenocarcinoma: A Propensity Score-Matched Analysis.
2	79	0.04	2017	Tumor Spread through Air Spaces Affects the Recurrence and Overall Survival in Patients with Lung Adenocarcinoma &gt;2 to 3 cm.
3	76	0.01	2015	Tumor Spread through Air Spaces is an Important Pattern of Invasion and Impacts the Frequency and Location of Recurrences after Limited Resection for Small Stage I Lung Adenocarcinomas.
4	71	0.02	2016	Spread through air spaces is a predictive factor of recurrence and a prognostic factor in stage I lung adenocarcinoma.
5	63	0.02	2017	Spread through Air Spaces (STAS) Is an Independent Predictor of Recurrence and Lung Cancer-Specific Death in Squamous Cell Carcinoma.
6	61	0	2015	Prognostic Impact of Intra-alveolar Tumor Spread in Pulmonary Adenocarcinoma.
7	60	0	2017	Semiquantitative Assessment of Tumor Spread through Air Spaces (STAS) in Early-Stage Lung Adenocarcinomas.
8	55	0.03	2017	Prognostic Impact of Margin Distance and Tumor Spread Through Air Spaces in Limited Resection for Primary Lung Cancer.
9	55	0.02	2018	Significance of Spread Through Air Spaces in Resected Pathological Stage I Lung Adenocarcinoma.
10	52	0.03	2019	Limited Resection Is Associated With a Higher Risk of Locoregional Recurrence than Lobectomy in Stage I Lung Adenocarcinoma With Tumor Spread Through Air Spaces.

**Figure 6:**
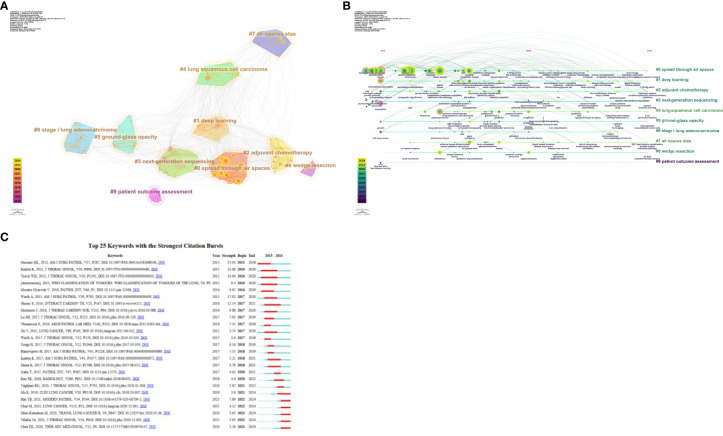
**(A)** Co-citation literature clustering analysis over the past decade. **(B)** Timeline of co-citation literature clusters. **(C)** Top 25 references by burst strength from 2015 to 2024.

**Figure 6A** displays the clusters generated from co-cited references based on keywords: #0 radiomics, #1 prognosis, #2 recurrence, #3 squamous cell carcinoma, #4 bronchial washing, #5 wedge resection, #6 gross handling, #7 lung cancer, #8 TNM classification, #9 patient outcome assessment, #10 regression analysis, #11 molecular pathology, #12 morphology, and #13 cribriform pattern. The clusters are ordered by size, with smaller ordinal values indicating larger clusters. The density of the lines connecting the clusters reflects their interrelationships. These clusters illustrate a comprehensive research area primarily focused on the diagnosis, prognosis, treatment, and pathology of lung cancer.

By analyzing these clusters, it becomes evident that current research hotspots include imaging analysis, prognostic assessment, recurrence prediction, pathological features, and their clinical applications. This integrated perspective aids in understanding the diversity of research and future research directions. As shown in the timeline of co-cited literature clusters in **Figure 6B**, #0 radiomics is the largest cluster in this study. Additionally, #1 prognosis, #2 recurrence, and #13 cribriform pattern have emerged as prominent research topics in recent years.

**Figure 6C** shows the top 25 most cited references. The most robust citation burst occurred for the 2015 article “Tumor Spread through Air Spaces is an Important Pattern of Invasion and Impacts the Frequency and Location of Recurrences after Limited Resection for Small Stage I Lung Adenocarcinomas,” which had a burst strength of 21.66. This article posits that STAS is a significant risk factor for recurrence after limited resection of small lung adenocarcinomas. STAS should be formally recognized as a mode of invasion for lung adenocarcinoma.

### Co-occurrence and overlay analysis of keywords

**Figure 7:**
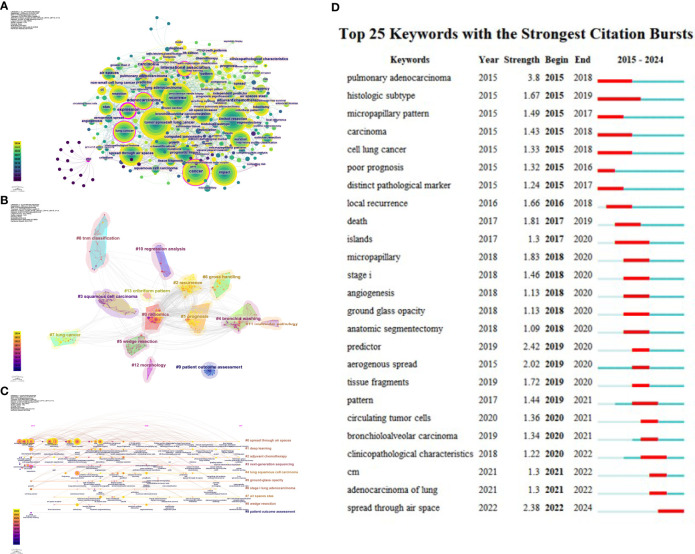
**(A)** Co-occurrence map of keywords. **(B)** Keyword clustering. **(C)** Timeline of keyword clusters. **(D)** Top 25 keywords by burst strength from 2015 to 2024.

Using CiteSpace to analyze keywords, **Table 8** lists the top ten most frequently occurring keywords, with “recurrence” being the most frequent, followed by “tumor spread” and “limited resection.” **Figure 7A** presents the co-occurrence map of keywords, where “expression,” “international association,” “lung cancer,” “carcinoma,” “end,” “cancer,” and “adenocarcinoma” have centrality values greater than 0.1, highlighted in the network with purple outer circles.

**Table 8:** Top 10 Keywords with Frequency from 2015 to 2024.

Sequence	count	centrality	year	keywords
1	120	0.03	2016	recurrence
2	111	0.01	2017	tumor spread
3	92	0.07	2016	limited resection
4	91	0.13	2015	cancer
5	78	0.05	2016	impact
6	78	0.09	2015	classification
7	74	0.03	2016	lung adenocarcinoma
8	70	0.02	2015	spread through air spaces
9	55	0.17	2015	lung cancer
10	48	0.04	2018	prognostic impact

Keyword clustering refers to grouping similar thematic keywords into multiple categories, while co-occurrence analysis helps identify relationships among various topics within a discipline. When analyzing the structure of scientific knowledge networks, keyword co-occurrence and clustering analysis aid in understanding the primary research hotspots of the field. **Figure 7B** illustrates the keyword clustering. Using the logarithmic likelihood ratio (LLR) algorithm, keywords were divided into 10 clusters, including #0 spread through air spaces, #1 deep learning, #2 adjuvant chemotherapy, #3 next-generation sequencing, #4 lung squamous cell carcinoma, #5 ground-glass opacity or ratio, #6 stage I lung adenocarcinoma, #7 air spaces STAS, #8 wedge resection, and #9 patient outcome assessment. **Figure 7C** presents the timeline of keyword clusters, with colors ranging from purple to yellow, indicating the passage of time from distant to recent. The keyword “patient outcome assessment” appeared relatively early, while “robotics” emerged later. Keywords such as “machine learning,” “growth pattern,” “bronchial cytology,” and “genomic features” have emerged in recent years. These changes signal a revolution in research hotspots over the past decade.

Keyword burst detection was conducted based on the keyword co-citation network. The top 25 most cited keywords in lung cancer STAS were analyzed. As shown in **Figure 7D**, the blue line represents the timeline, while the red segments on the blue timeline indicate burst detection, representing the start year, end year, and duration of the burst. It was found that “pulmonary adenocarcinoma” (3.8) had the most robust citation burst, followed by “predictor” (2.42), “spread through air space” (2.38), “anatomic segmentectomy” (2.02), “micropapillary” (1.83), and “death” (1.81). Based on the start times of appearance, it can be observed that “pulmonary adenocarcinoma,” “histologic subtype,” “micropapillary pattern,” “carcinoma,” “cell lung cancer,” “poor prognosis,” and “distinct pathological marker” emerged earlier and were subjects of early attention. Currently, “predictor,” “aerogenous spread,” “tissue fragments,” “pattern,” “circulating tumor cells,” “bronchioloalveolar carcinoma,” “clinicopathological characteristics,” “CM,” “adenocarcinoma of the lung,” and “spread through air space” represent the forefront of research in lung cancer STAS.

## Discussion

Since the World Health Organization identified STAS as a new mode of invasion for lung adenocarcinoma in 2015, the number of articles related to STAS has sharply increased in recent years. This study used the R package bibliometrix and visualization software CiteSpace and VOSviewer to analyze the bibliometric data related to lung cancer STAS. We systematically and objectively described the development trends and future research hotspots in this field to help scholars quickly understand the current state of research and provide valuable guidance for topic selection. The first part of this study analyzed publication trends, countries, institutions, authors, and journals. Subsequently, a clustering analysis of keywords was performed to identify research hotspots in the field.

According to the results on publication trends, the number of publications related to lung cancer STAS has rapidly increased over the past six years, accounting for 86.8% of the total published papers. However, the overall number of publications on lung cancer STAS remains relatively low, indicating that this field is still in its early stages. The R² value of 0.9597 suggests that the literature output in this field is expected to proliferate, with more countries and researchers likely to engage in this study area. China and Japan are the two countries with the highest number of publications on lung cancer STAS. Although China has the highest number of papers, the average citation count is lower, indicating a need to improve the quality and impact of research. Canada, despite having only four publications, has the highest average citation count. The most prolific author is Kadota K, who demonstrates a strong influence and a core position within the academic network. He is a prominent scholar in lung cancer and STAS research, making significant contributions to understanding the patterns of STAS dissemination.

Clustering based on co-cited references is developed by centering on the citation relationships among literatures, reflecting the correlation of research in knowledge inheritance and citation network. Keyword-based clustering is mainly divided according to the keywords contained in the literature itself, which more directly reflects the similarity of the topic content of the literature. By comparing the two, it is found that the cluster mainly focuses on the diagnosis, prognosis, treatment and pathology of lung cancer. We divide these clusters into four clusters: pathological research of STAS, potential mechanisms, prognostic assessment, Imaging analysis and model prediction. We will discuss the research hotspots and trends within each cluster in detail.

### Cluster 1: Pathological research on lung cancer STAS

In 1980, Kodama et al. (8) first proposed the concept in a report of a lung cancer case where homologous cancer cells were found in the airways outside the primary lesion. In 2013, Onozato et al. (9) observed islands of tumor cells in lung adenocarcinoma that were not connected to the primary tumor mass. It was not until 2015 that the World Health Organization (WHO) officially defined STAS, describing it as the spread of micropapillary clusters, solid nests, or individual tumor cells within the alveolar spaces surrounding the primary tumor, categorized into three pathological patterns: micropapillary structures, solid nests of tumor cells, and single tumor cells (3). In the same year, Warth et al. (6) classified STAS into localized (within three alveolar spaces from the primary tumor) and extensive (beyond three alveolar spaces) types. In 2017, Uruga et al. (10) conducted a semi-quantitative assessment of STAS, dividing it into no STAS, low STAS (1-4 cells), and high STAS (≥5 cells), providing essential grounds for risk stratification and subsequent research on lung adenocarcinoma STAS.

STAS is more commonly found in more aggressive forms of lung cancer, such as micropapillary, papillary, and solid types (10). Its prevalence is higher in moderately to poorly differentiated lung cancers than in well-differentiated lung cancers (11). Furthermore, STAS is associated with lymph node metastasis and invasion of the pleura and neurovascular structures (12, 13).

### Cluster 2:Potential mechanisms of lung cancer STAS

Since the concept of STAS was introduced, numerous related studies have been conducted; however, the specific mechanisms of its occurrence and development remain unclear, with only some preliminary findings and hypotheses available. Current research indicates that the occurrence of STAS is closely related to epithelial-mesenchymal transition (EMT), the tumor microenvironment, and specific lung cancer-associated genes. The interactions between cells and between cells and the extracellular matrix during the EMT process lead to reorganization (14). STAS is associated with low expression of epithelial cadherin and high expression of vimentin and Ki-67 (15). Additionally, MMP-7 promotes tumor cell migration by degrading the extracellular matrix, subsequently leading to STAS (16). High expression levels of fibroblasts and macrophages in the tumor microenvironment are significantly associated with STAS (17). NRG1 inhibits the occurrence of STAS by reducing tumor cell invasiveness and migratory capacity through the suppression of the AKT and ERK1/2 signaling pathways (18). Activation of PGC-1α regulates fatty acid oxidation and mitochondrial activity in CTLs, thereby enhancing antitumor capacity and inhibiting the spread and invasion of STAS (19). Genetic testing has shown that STAS is more common in lung cancer patients with wild-type EGFR and ALK rearrangements (20), while it is not associated with KRAS mutations (21). Despite multiple related factors, the specific mechanisms underlying STAS require further investigation. Additionally, the tumor microenvironment, signaling pathways, and molecular abnormalities play a critical role in tumor invasion and dissemination, as well as in other diseases (22–24).

### Cluster 3: Prognostic assessment of lung cancer STAS

STAS can be found in various subtypes of lung cancer, particularly in adenocarcinomas (ADC), characterized by a predominance of micropapillary growth patterns (25), and it significantly impacts the prognosis of early-stage ADC. Studies show that STAS-positive patients have a markedly higher risk of local or distant recurrence in stage I ADC compared to STAS-negative patients, with better outcomes observed in those undergoing lobectomy rather than sublobar resection (26, 27). STAS is also an independent risk factor for recurrence in stage I solid ADC patients and holds prognostic value for ADC patients at all stages (21, 28). The presence of STAS and its distance are critical factors influencing the prognosis of lung adenocarcinoma. The greater the distance from the tumor margin to the farthest STAS, the higher the risk of recurrence and mortality, with each 1-mm increase in distance associated with a 1.26-fold increase in mortality risk (29). STAS is a significant negative prognostic factor in lung adenocarcinoma patients, particularly in those undergoing limited resection, where it significantly increases the risk of recurrence and reduces survival rates. For STAS-positive patients, more extensive surgical resection or closer postoperative surveillance and treatment may be required (30).In lung squamous cell carcinoma, the rate of STAS positivity is 30%, and it is associated with pathological staging, lymph node metastasis, and tumor budding, serving as an independent predictor of recurrence-free survival (4, 31). Research on lung neuroendocrine tumors (NETs) indicates that the presence of STAS is associated with early distant metastasis and specific mortality rates, and it is considered an independent adverse prognostic factor in both large-cell lung carcinoma and small-cell lung carcinoma (5).In summary, STAS positivity significantly correlates with shortened recurrence-free survival (RFS) and overall survival (OS). Consequently, the preoperative prediction of STAS in lung cancer patients has become a current research hotspot, with many scholars aiming to predict STAS preoperatively using imaging techniques.

### Cluster 4: Imaging analysis and model prediction of lung cancer STAS

STAS (aerogenous dissemination) is associated with various CT features, such as tumor margins, speculation, and lobulation (11, 32), reflecting the infiltrative growth of the tumor. Studies have shown that the maximum diameter of the nodule (32) and the maximum diameter of the solid component (33) are significantly related to STAS. The solid component ratio (CTR) is considered an independent risk factor for STAS (34, 35), particularly in lung adenocarcinoma ≤3 Cm, where it can effectively predict STAS, although with low specificity (36). A solid component proportion greater than 50% in solid and part-solid nodules is an important predictive factor (37). Although STAS can also be found in pure ground-glass nodules, the incidence is only 2.42% (36).

With the application of AI in imaging, radiomics has rapidly developed, utilizing high-throughput extraction of imaging information to analyze and mine high-dimensional data, aiding radiologists in diagnosis (38). Many researchers have begun to explore the application of radiomics in predicting STAS in non-small cell lung cancer (NSCLC). Chen et al. (39) constructed a predictive model for 233 patients with stage I lung adenocarcinoma, achieving AUCs of 0.63 and 0.69 for internal and external validation, demonstrating CT radiomics’s potential. Han et al. (40) applied a logistic regression model in stage IA patients, reaching AUC values of 0.812 and 0.850. Jiang et al. (41) used a random forest classifier, obtaining an AUC of 0.754. Zhuo (32) and Qi et al. (34) found that comprehensive models combining peritumoral regions with other clinical parameters were more effective. Wang et al. (42) identified that a radiomics model within 5 mm of the tumor in stage IA NSCLC yielded the best results, emphasizing the relationship between STAS and tumor invasiveness.

Deep learning features have become an essential complement to traditional radiomic features in medical imaging, with features extracted using convolutional neural networks (CNNs) significantly improving model performance in clinical tasks. Tao et al. (43) investigated the application of a three-dimensional convolutional neural network (3D CNN) model based on contrast-enhanced CT in predicting the spread through air spaces (STAS) status of non-small cell lung cancer (NSCLC). The results demonstrated that the AUC of the 3D CNN model reached 0.93 in the training set and 0.80 in the validation set, which was significantly superior to that of the traditional radiomics model and the computer vision (CV) model (with AUCs of 0.79 and 0.77 respectively). The 3D CNN model is capable of automatically extracting complex three-dimensional features from CT images, overcoming the limitations of manual feature selection, and exhibits higher robustness in dealing with changes in CT scanning parameters and segmentation errors, thus providing a more reliable basis for preoperative surgical planning and enhancing the accuracy of STAS prediction. Jin et al. (44) proposed a dynamic dual-delta model that combines deep learning and radiomics features to efficiently predict the spread of lung cancer through air spaces (STAS) by dynamically capturing tumor changes on preoperative CT. It achieved an AUC of 0.94 in internal validation and 0.84 in external validation, demonstrating excellent predictive performance and cross-center robustness, and providing important support for preoperative decision-making and precise treatment of lung cancer. Wang et al. (45) developed a CT-based deep learning model, SE-ResNet50, for predicting the spread through air spaces (STAS) status of solid or part-solid lung adenocarcinoma (LUAD). By integrating super-resolution (SR) technology and an attention mechanism, SE-ResNet50 attained a prediction accuracy of 82.2% (compared with 72.2% of ResNet50) and an AUC of 0.806 (compared with 0.695) in external validation, with sensitivities and specificities of 61.5% and 90.6% respectively. Through dynamically adjusting feature weights, this model enhanced its ability to capture key features and had a higher predictive efficiency compared with the radiomics method, providing precise support for preoperative assessment and surgical decision-making. Lin et al. (46) developed a deep learning model based on CT images (STAS-DL) that can effectively predict the spread of tumors through air spaces (STAS) in ground-glass-dominant lung adenocarcinoma. The AUC was 0.82 and the prediction accuracy was 74% in the test set, which was better than that of the traditional radiomics model (AUC = 0.78) and the C/T ratio model (AUC = 0.77). By introducing the “solid component gating” (SCG) channel, the model focused on the solid regions of the tumor, increasing the sensitivity to 79% and the specificity to 73%, providing important support for preoperative decision-making in lung adenocarcinoma patients with a tumor size less than 3 cm and a C/T ratio lower than 0.5.Dou et al. (47) found through research that the spread through air spaces (STAS) is closely related to the poor prognosis of lung adenocarcinoma patients, and accurate preoperative prediction of the STAS status is of great significance for optimizing surgical plans. Their team proposed a MultiCL model based on a deep neural network (DNN), which fully exploited the three-dimensional features in CT images through supervised contrast learning and fine-tuning strategies, reaching AUC values of 0.8434 in the test set and 0.7686 in the external validation set respectively, which was significantly better than the external validation performance of the traditional radiomics model (AUC = 0.7796). This study indicates that deep learning models can effectively capture the invisible features in CT images, providing important support for non-invasive preoperative detection of STAS and personalized surgical decision-making, especially having important clinical significance in guiding STAS-positive patients to avoid sublobar resection. Additionally, deep learning has shown excellent performance in predicting STAS in early lung adenocarcinoma. These studies have demonstrated that deep learning models possess significant potential in predicting STAS preoperatively. They can predict STAS more accurately, which can improve the assessment of patients’ conditions and risk stratification, be more conducive to accurately guiding patients’ surgical decision-making, and contribute to the formulation of more personalized treatment regimens. Additionally, by enhancing the diagnostic accuracy and consistency of STAS, the possibilities of misdiagnosis or missed diagnosis may be reduced, thereby improving patients’ long-term survival rates and promoting more efficient diagnostic methods.

PET-CT has shown significant value in predicting STAS and is associated with high tumor metabolism. Research has found that a ratio of tumor metabolic volume (MTV) to tumor CT volume (CTV) greater than 1 can predict STAS (48). STAS-positive tumors’ standardized uptake value (SUV) is significantly higher than negative tumors (12). Multi-parametric analysis of spectral CT may also provide significant value for predicting STAS.

### Limitations of the study

This study has several limitations, similar to previous bibliometric studies. First, only the WOSCC database was selected and the databases in other languages were ignored, which may have resulted in the omission of some data. Second, inconsistencies in the formatting of author or institution names may result in the dispersion of research counts. Additionally, this study cannot ensure that all included publications fully meet the thematic relevance search criteria. Furthermore, the relatively small number of related papers and the fact that the literature search was conducted up to August 2024 limit the comprehensiveness of the included literature for that year. Nevertheless, the analysis in this study still provides valuable insights into the field’s current state.

## Conclusion

In conclusion, research on lung cancer STAS is still in its early stages, with the main areas of investigation focusing on pathology, mechanisms, prediction, and prognosis. STAS represents a novel invasive pattern and is of crucial importance for the diagnosis, treatment, and prognosis of lung cancer patients. The precise prediction of preoperative STAS by deep learning radiomics is helpful in formulating more personalized treatment plans for lung cancer patients, thereby improving their long-term survival rates. Moreover, it is necessary to train clinicians to apply these deep learning models in clinical practice. This bibliometric study provides a summary and insights into the development trends in lung cancer STAS research, emphasizing the discipline’s current state. This perspective helps researchers identify hotspots and frontiers in the field and new research directions.

## Data Availability

The original contributions presented in the study are included in the article/supplementary material. Further inquiries can be directed to the corresponding author.
